# Algorithm for Reducing Overall Biological Detriment Caused by PET/CT: an Age-Based Study

**DOI:** 10.1007/s13139-023-00788-4

**Published:** 2023-02-04

**Authors:** Marco Spadafora, Pasqualina Sannino, Luigi Mansi, Ciro Mainolfi, Rosario Capasso, Eugenio Di Giorgio, Salvatore Fiordoro, Serena Imbimbo, Filomena Masone, Laura Evangelista

**Affiliations:** 1 Nuclear Medicine Unit, Ospedale del Mare, Naples, Italy; 2 CIRPS, Interuniversity Research Center for Sustainability, Rome, Italy; 3 IOS–Medicina Futura, Acerra, Naples, Italy; 4 Department of Advanced Biomedical Sciences, University of Naples Federico II, Naples, Italy; 5 Nuclear Medicine Unit, Department of Medicine (DIMED), University of Padua, Via Giustiniani 2, 35128 Padua, Italy

**Keywords:** PET, FDG, Radiation risk, Effective dose, Additional cancer risk

## Abstract

**Purpose:**

This study is to use a simple algorithm based on patient’s age to reduce the overall biological detriment associated with PET/CT.

**Materials and Methods:**

A total of 421 consecutive patients (mean age 64 ± 14 years) undergoing PET for various clinical indications were enrolled. For each scan, effective dose (ED in mSv) and additional cancer risk (ACR) were computed both in a reference condition (REF) and after applying an original algorithm (ALGO). The ALGO modified the mean dose of FDG and the PET scan time parameters; indeed, a lower dose and a longer scan time were reported in the younger, while a higher dose and a shorter scan time in the older patients. Moreover, patients were classified by age bracket (18–29, 30–60, and 61–90 years).

**Results:**

The ED was 4.57 ± 0.92 mSv in the REF condition. The ACR were 0.020 ± 0.016 and 0.0187 ± 0.013, respectively, in REF and ALGO. The ACR for the REF and ALGO conditions were significantly reduced in males and females, although it was more evident in the latter gender (all *p* < 0.0001). Finally, the ACR significantly reduced from the REF condition to ALGO in all three age brackets (all *p* < 0.0001).

**Conclusion:**

Implementation of ALGO protocols in PET can reduce the overall ACR, mainly in young and female patients.

## Introduction

The clinical role of positron emission tomography (PET) and computed tomography (CT) has been firmly established, but the scan exposes patients to a considerable amount of radiation [1, 2]. In Western countries, medical diagnostic radiation has been the fastest-growing component of radiation exposure for the general population [3]. New PET and CT hardware and software are continuously being developed and now include the use of digital detector, image reconstruction algorithms, and artificial intelligence [4–6]). Novel acquisition protocols that modify injected doses and/or acquisition times have also been proposed to optimize PET studies [4–8].

A standard 18F-fluorodeoxyglucose (FDG) PET/CT scan can be associated a total dose of 21.64 ± 5.20 mSv, an average radiation dose of 8.19 ± 0.83 mSv and 13.44 ± 5.14 mSv for the PET and CT components, respectively [9]. Based on a recent survey made in Korea in more than 30 hospitals, effective doses for torso FDG PET/CT were significantly different in patients with a diverse body weight; indeed, it was higher in 70-kg and 85-kg patients than those with a 55 kg of body weight. Moreover, the CT effective dose accounted for more than 43% of the total effective dose. Therefore, the promotion of programs able to reduce CT dose and the personalization of FDG injected dose should be the goals for decreasing unnecessary radiation exposure [10].

Integrating PET with magnetic resonance imaging (MRI) instead of CT scans lowers the exposure to radiation, about 50% [11]. Hybrid PET/CT scanning can also be done with a lower exposure, however, by reducing the dose of radiation for the CT component of the hybrid tool [12, 13]. Moreover, also the introduction of PET/MR and new scanners, such as digital and total-body PET/CT, could significantly improve the management of radiation protection, mainly in pediatric patients.

The fundamental role of radiation-induced DNA damage is the induction of mutations and chromosome aberrations, with the risk of cancer induction. This stochastic risk, on this basis of linear no-threshold hypothesis, may occur also at low-dose-rate exposures. For an exposure greater than 20 mSv per year, the ICRP states that efforts should be made to reduce doses. While the linear no-threshold hypothesizes that a given dose, even lower than 100 mSv, produces a proportional increase of cancer risk, non-linear responses at low dose may produce an up-regulation of defenses with an adaptative protection against tumor. However, the current uncertainties about these mechanisms do not allow for practical implications. It is not easy to quantify the cancer risk of low-dose radiation. Several groups have reported that medical exposure is associated with a higher risk of developing secondary cancers later in life. This is a particular concern for younger patients because they are more susceptible to the harmful effects of radiation than adults, and their life expectancy is longer [14–17]. In the ongoing debate on the incremental risk to individuals exposed to low diagnostic doses of radiation, it is nonetheless inappropriate to discuss the potential risks while ignoring the corresponding benefits [18–20]. Prior study suggest that submitting radiological imaging to the linear no-threshold hypothesis (LNTH) or the ALARA (as low as reasonably achievable) principle is “a non sequitur of non-trivial proportion,” on the grounds that fear-driven imaging could hinder rapid diagnoses, life-saving treatments, and quality-of-life improvements, with shorter hospital stays and lower costs [19]. Other authors claim instead that such a linearity assumption is not necessarily the most conservative approach and it probably leads to some radiation-induced cancer risks being underestimated and to others being overestimated [17]. Regulatory agencies such as the International Commission on Radiological Protection (ICRP) and Euratom have nonetheless reiterated the need to apply the basic principles of radiation protection to the dangers deriving from medical exposure [21, 22]. According to a principle of optimization, individual doses should be kept at the lowest feasible level compatible with the diagnostic information obtainable. National laws implementing these international directives (such as 2013/59 Euratom) also establish severe sanctions—including criminal penalties—for any misconduct. This means that, regardless of the scientific theories on the risks of exposure, the pressure of regulatory agencies has made efforts to contain individual exposure—with no loss of diagnostic detail—mandatory, not a matter of choice.

Current guidelines for PET imaging recommend an administered radiotracer (i.e., FDG) activity based on a patient’s weight and the technical performance of the scanner [23, 24]. Beyond childhood and adolescence, age is no longer a factor influencing tracer dosage. Age at the time of exposure and gender are nonetheless two key factors, which can raise the risk of radiation detriment by a factor of more than 2 and up to 6, respectively. This was underscored in the recent ICRP Strategic Plan that recommends considering a sex- and age-nominal risk and avoiding average values, when calculating radiation detriment [25–28].

The aim of the study was to evaluate the effects on the theoretical risk of cancer by modifying both the injected radiopharmaceutical dose and acquisition scan-time based on a patient's age, during a daily PET/CT study session.

## Materials and Methods

### Study Design

This was a single-center, retrospective cohort study on 421 patients who underwent FDG PET/CT in 43 consecutive FDG PET/CT study sessions. Patients aged > 18 years scheduled for whole-body FDG PET/CT, who signed to give their informed consent, were included. Pediatric patients and clinical emergencies were excluded.

### PET Image Acquisition Protocol

PET imaging was done on a Siemens Biograph mCT, PET/CT system using a standard comparable protocol with integrated 3D mode PET/CT systems, scanning from the base or top of the skull to the mid-thigh (wb-PET/CT), and starting 60 min after administering the tracer. The acquisition PET/CT mode was a continuous-motion of the patient table by flow modality, instead of multi-bed position-based planning. The sliding speed of the bed was 0.7 mm/sec. An iterative method, with 3 iterations and 21 subsets, was used for image reconstruction. Attenuation correction was done using CT images. CT and PET images were matched and fused into transaxial, coronal, and sagittal images. All patients fasted for at least 6 h prior to imaging, and blood glucose levels were < 180 mg/dL at the time of tracer injection.

### Data Collected and Original Algorithm

The data recorded included patients’ age and sex, scan time, FDG dose, radiation exposure (expressed as the effective dose [ED] in mSv), and additional cancer risk (ACR). The ED was calculated according to the ICRP 103 [20]. The ACR was assessed on risk models described in the Biological Effects of Ionizing Radiation (BEIR) VII report [29]. These parameters were calculated in a reference condition (REF), and after applying an original algorithm (ALGO) that modified the PET dose and scan-time without changing the CT parameters such as kilovolts and milliampere. Based on the time-dose equation, the ALGO inversely modified the FDG dose and PET scan time by ± 20% to obtain the same counting statistic for each PET study. In ALGO, as shown in Table 1, in the three younger patients of each 43 consecutive FDG PET/CT diagnostic sessions, the dose of the tracer was reduced by 20%, where it was increased by the same percentage the scan time. Conversely, in the three older patients the dose of the tracer was increased by 20%, where it was reduced by the same percentage the scan time. A practical example of ALGO is the following. In a session of PET/CT examinations, three young patients received a 20% reduced dose of FDG (from 250, 280, and 310 MBq to 200, 224, and 248 MBq, respectively). In order to obtain an adequate counting statistic for each PET study, the scan time was increased by 20%. Conversely, in three old patients scheduled in the same day, the FDG doses were increased by 20% (i.e., from 250, 280, and 310 MBq to 300, 336, and 372 MBq, respectively), and therefore, the scan time was reduced by 20%. This modification of both FDG dosage and acquisition time, according to the age patients was called ALGO. Moreover, for each PET session, the ACR was also assessed in both sexes and for three age brackets (18–29, 30–60, and 61–90 years) regardless of gender.

**Table 1:** Demographic data and FDG-PET parameters in an illustrative single study session

GENDER	Age (y)	Weight (kg)	DOSE	DOSE ALGO	Scan time	Scan time ALGO	ED REF (mSv)	ACR REF	ACR ALGO
M	19	87	362	289	1211	1465	5.792	0.0687	0.0549
F	29	75	268	214	1113	1335	4.288	0.0592	0.0473
M	51	70	283	226	1165	1398	4.528	0.0223	0.0178
M	63	88	277	277	1217	1217	4.432	0.0157	0.0157
F	67	55	241	241	956	956	3.856	0.0146	0.0146
F	70	90	271	271	1124	1124	4.336	0.0149	0.0149
M	76	70	352	352	1193	1193	5.632	0.0139	0.0139
M	77	55	230	276	1188	950	3.68	0.0089	0.0106
M	79	64	202	242	1338	1070	3.232	0.0074	0.0088
F	81	58	247	296	1315	1052	3.952	0.0093	0.0111

*F* female, *M* male; the dose refers to the FDG in MBq; scan time in seconds; *ED* effective dose in mSv

### Statistical Analysis

Data were expressed as mean ± standard deviation, or as median (range). Differences between continuous data were assessed using Student’s paired *t*-test. The Microsoft Excel by Windows was used for the statistical analysis. A *p* < 0.05 was set for the significance.

## Results

In the 43 PET/CT session, a total of 421 patients (57% males; mean age 64 ± 14 years, range 18–90 years) were retrospectively analyzed. The median age of the patients was 66 years. The mean number of patients per session was 9.8 (range 7–13). The mean weight of the patients was 72.3 ± 14.2 kg. The mean dose of FDG administered was 285.7 + 57.3 MBq (range 185–399 MBq), equating to 3.95 MBq/kg. The mean PET scan time was 1191 ± 198 s. The ALGO changed these dose and time parameters so that younger patients had a 20% lower dose and a 20% longer scan, while older patients had a 20% higher dose and a 20% shorter scan. By itself, this approach does not significantly change the overall duration of the session, or the amount of tracer injected (Table 1).

**Fig. 1:**
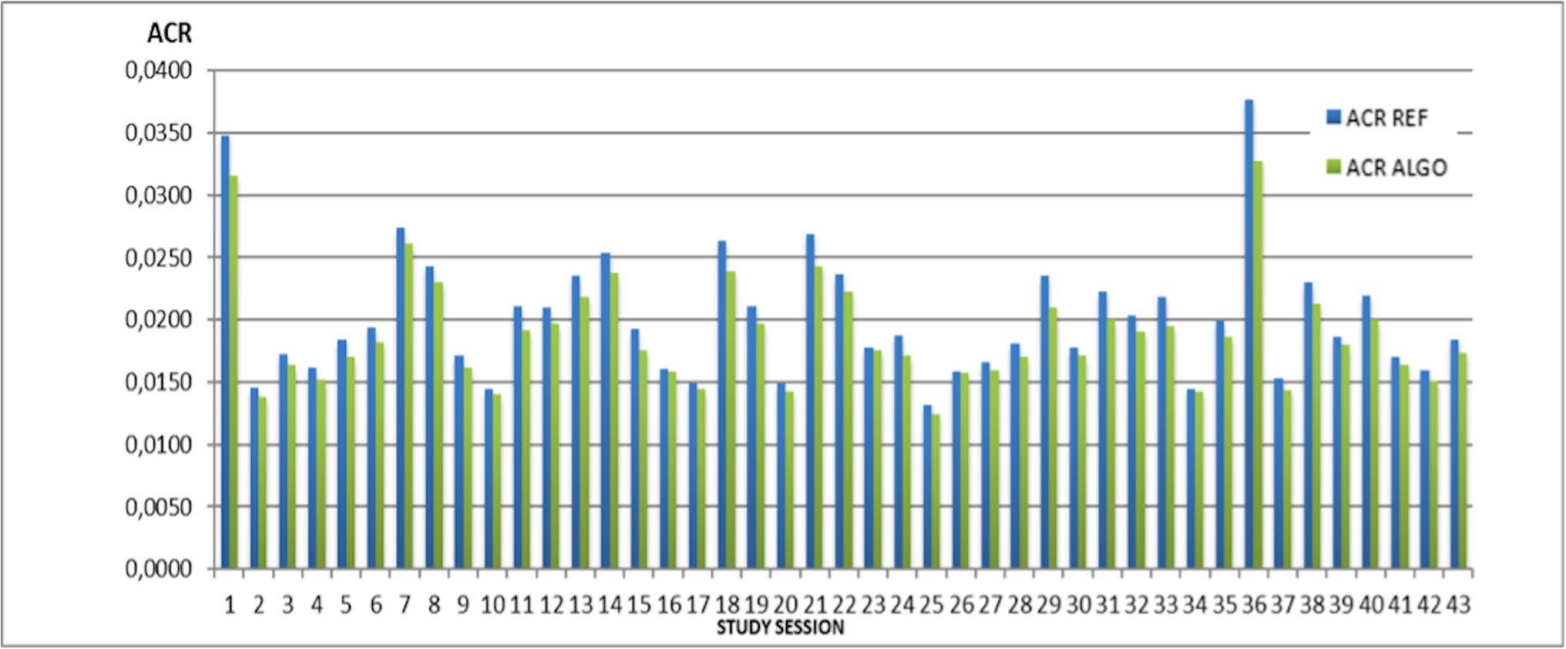
Comparison among ACR REF and ACR ALGO, in study population for each of the 43 study sessions

**Fig. 2:**
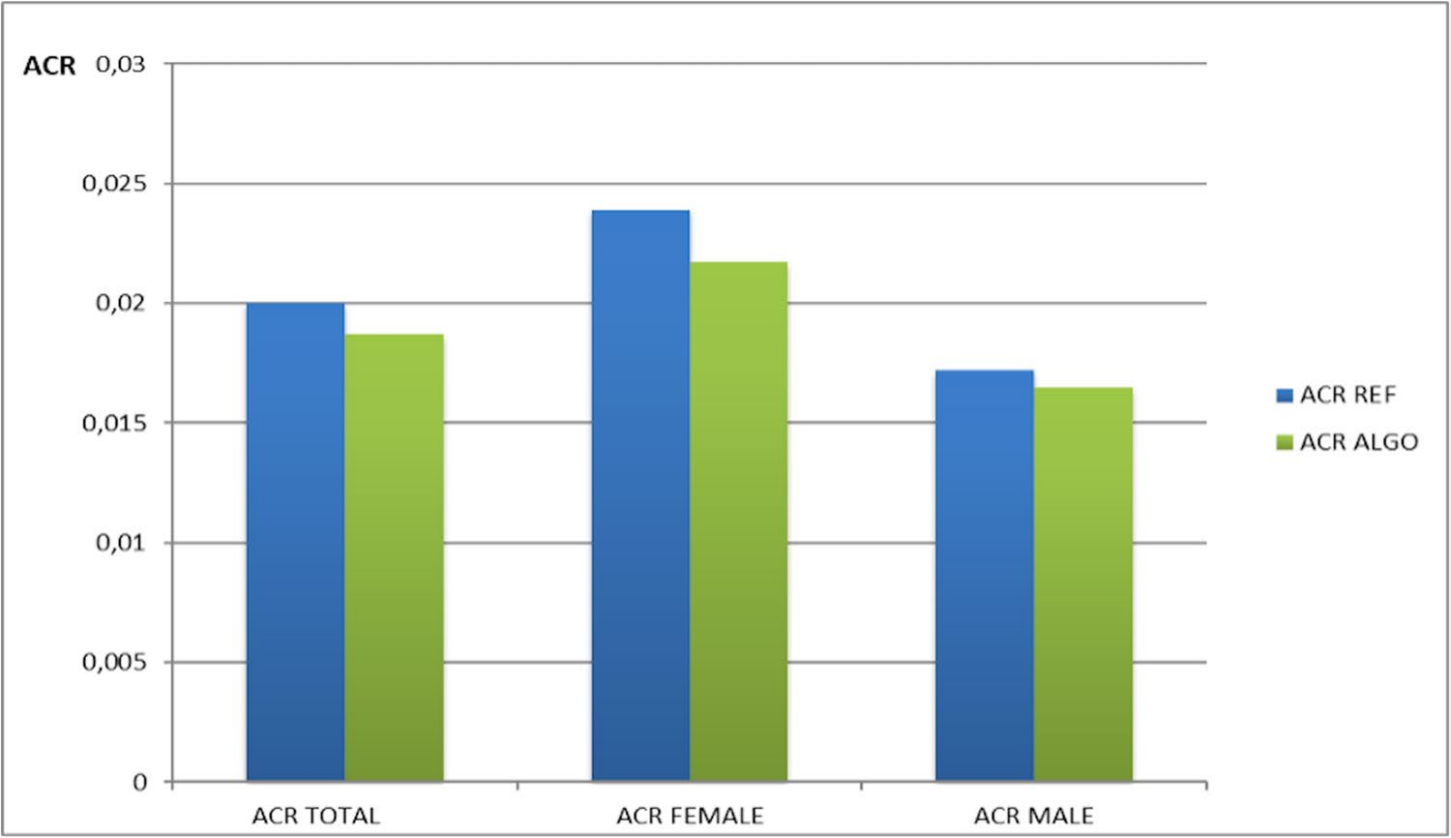
Comparison among ACR REF and ACR ALGO, in study population and sex categories. All comparison *p* < 0.0001)

Demographic data and ACR values for each of the 43 study sessions are reported in Table 2. Figure 1 shows the distribution of the values for ACR REF and ACR ALGO, in study population in each study session. Figure 2 shows the distribution of data for ACR REF and ACR ALGO in all study population and sex categories. Mean values of ED and ACR in each category are reported in Table 3.

**Table 2:** Demographic data and ACR values for each of the 43 consecutive study sessions

PET/CT session	Gender	Age min	Age max	Mean ACR REF	Mean ACR ALGO	ACR decrease ALGO vs REF
1	6F, 3M	18	70	0.0347	0.0315	9.25%
2	1F, 9M	43	85	0.0145	0.0138	5.16%
3	6F, 3M	49	78	0.0172	0.0164	5.12%
4	4F, 8M	51	82	0.0161	0.0152	5.51%
5	2F, 6M	48	85	0.0184	0.0170	7.45%
6	1F, 7M	40	84	0.0194	0.0182	6.24%
7	10F, 2M	31	77	0.0274	0.0261	5.03%
8	5F, 5M	36	78	0.0243	0.0230	5.22%
9	6F, 6M	46	84	0.0171	0.0161	5.83%
10	4F, 6M	54	90	0.0145	0.0140	3.47%
11	4F, 4M	28	84	0.0211	0.0192	9.07%
12	5F, 6M	43	76	0.0210	0.0197	6.20%
13	4F, 7M	37	88	0.0236	0.0218	7.66%
14	2F, 7M	28	75	0.0254	0.0237	6.46%
15	4F, 4M	34	85	0.0192	0.0176	8.54%
16	5F, 3M	21	75	0.0161	0.0159	1.34%
17	2F, 7M	57	80	0.0149	0.0145	2.53%
18	4F, 4M	18	75	0.0264	0.0238	9.57%
19	3F, 5M	26	83	0.0211	0.0197	6.45%
20	3F, 8M	55	84	0.0148	0.0142	4.27%
21	2F, 7M	20	75	0.0269	0.0243	9.47%
22	2F, 6M	24	74	0.0237	0.0223	5.82%
23	3F, 7M	47	81	0.0177	0.0175	0.97%
24	5F, 3M	42	84	0.0187	0.0172	8.19%
25	4F, 8M	48	85	0.0132	0.0124	6.06%
26	4F, 5M	54	73	0.0158	0.0157	0.69%
27	5F, 2M	53	83	0.0166	0.0159	4.04%
28	3F, 5M	38	81	0.0181	0.0170	5.79%
29	4F, 6M	19	81	0.0235	0.0210	10.61%
30	4F, 7M	30	82	0.0178	0.0171	4.04%
31	4F, 4M	31	84	0.0222	0.0200	10.18%
32	7F, 5M	32	79	0.0204	0.0191	6.50%
33	5F, 4M	17	86	0.0218	0.0195	10.50%
34	9M	54	77	0.0144	0.0143	1.23%
35	7F, 4M	19	82	0.0199	0.0186	6.36%
36	6F, 4M	50	76	0.0377	0.0327	13.03%
37	7F, 5M	40	89	0.0153	0.0144	5.90%
38	4F, 5M	17	78	0.0230	0.0213	7.30%
39	5F, 5M	19	79	0.0186	0.0179	3.78%
40	8F, 5M	17	81	0.0220	0.0200	9.03%
41	4F, 7M	46	88	0.0170	0.0164	3.58%
42	1F, 11M	44	81	0.0159	0.0151	5.43%
43	4F, 6M	46	84	0.0184	0.0173	5.77%

*F* female, *M* male; age was expressed in years (min = minimum, max = maximum), *ACR* additional cancer risk, *REF* reference

**Table 3:** Comparison among ACR REF and ACR ALGO, in study population and in individual categories of patients (all comparison *p* < 0.0001)

	ACR total (421 PT)	ACR female (43%)	ACR male (57%)	Age 18–29 (4%)	Age 30–60 (30%)	Age 61–90 (66%)
ACR REF	0.0200 ± 0.016	0.0239 ± 0.022	0.0172 ± 0.009	0.061 ± 0.022	0.0289 ± 0.020	0.0134 ± 0.004
ACR ALGO	0.0187 ± 0.013	0.0217 ± 0.017	0.0165 ± 0.007	0.0486 ± 0.018	0.024 ± 0.016	0.0144 ± 0.004

Values are expressed as average ± standard deviation

In the study population, the ED and ACR in the REF condition were 4.57 ± 0.92 mSv and 0.020 ± 0.016, respectively. After applying ALGO, the ACR decreased to 0.0187 ± 0.013 (*p* < 0.0001). The ACR for the REF and ALGO were 0.0239 ± 0.022 and 0.217 ± 0.017, respectively, in females (*p* < 0.0001), and 0.0172 ± 0.009 and 0.165 ± 0.007, respectively, in males (*p* < 0.0001). Compared with REF, ALGO led to a percentage reduction in the ACR of 9.2% in females and of 4.1% in males. After applying ALGO the mean reduction in the ACR versus REF was 6.5% (range 0.7–13.0%), (Table 4).

**Table 4:** Percentage reduction in the ACR ALGO compared with ACR REF, in study population and in individual categories of patients

	ACR total	ACR female	ACR male	ACR 18–29	ACR 30–60	ACR 61–90
ACR ALGO	− 6.5	− 9.2	− 4.1	− 20.3	− 17	+ 7.4

Values are expressed as % less than ACR REF

**Fig. 3:**
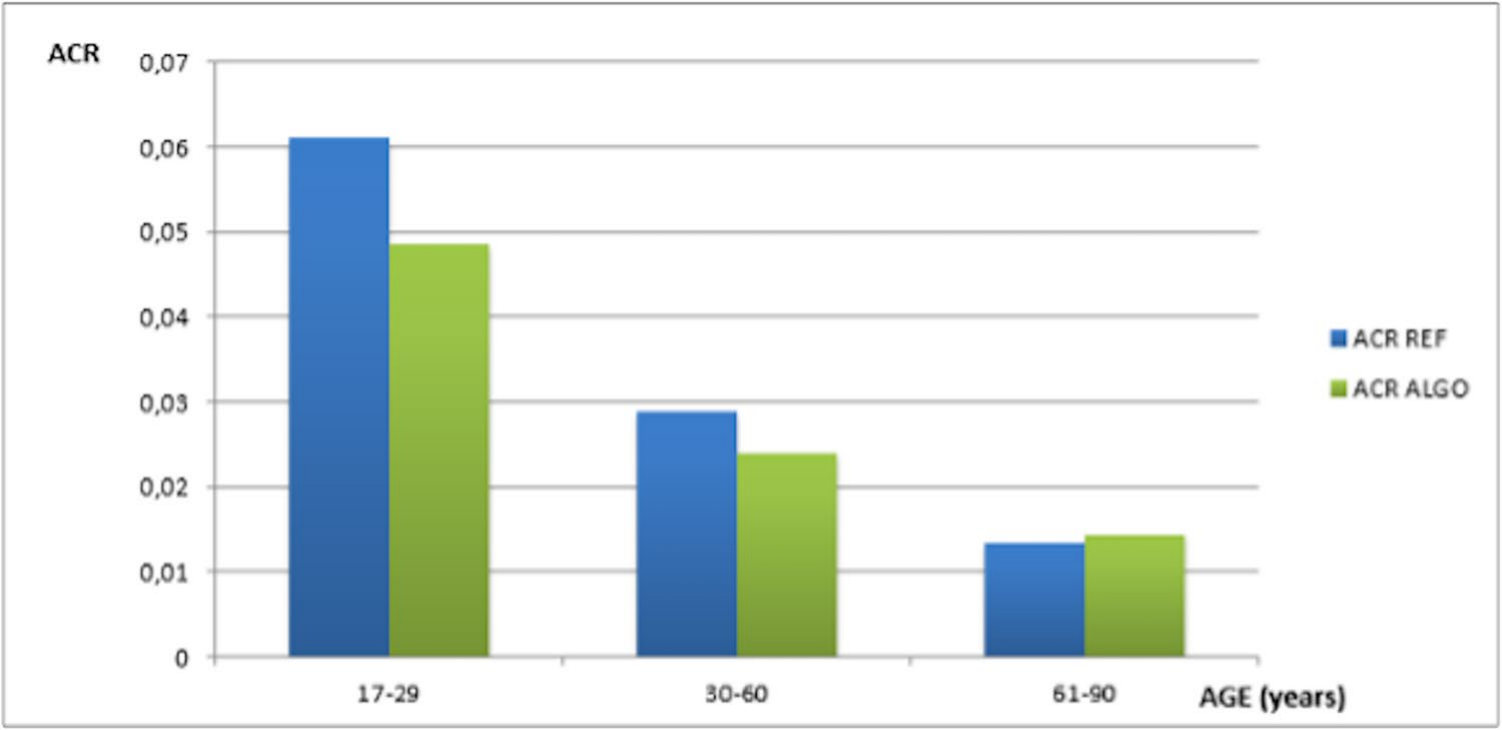
Comparison among ACR REF and ACR ALGO, in the three age categories. All comparison *p* < 0.0001

For the three age brackets (18–29 years old [17 pts, 4%]; 30–60 years old [126 pts, 30%]; and 61–90 years old [278 pts, 66%]), the ACR changed from 0.061 ± 0.022, 0.0289 ± 0.020, and 0.0134 ± 0.004 in the REF condition to 0.0486 ± 0.018, 0.0240 ± 0.016, and 0.0144 ± 0.004 for ALGO (all *p* < 0.0001) (Fig. 3). In these three age brackets, when ALGO was compared to REF, the changes in ACR were − 20.3%, − 17.0%, and + 7.4% (Table 4).

## Discussion

The main finding of this study is that a simple and original algorithm based on modifying PET radiopharmaceutical dose according to patients’ age significantly reduced the overall biological cancer risk of radiation detriment caused by PET/CT by up to 20%. The benefit was greatest for younger patients and females. It is surprising that among the factors considered for establishing the FDG dose, both age and sex of the patient are ignored. Indeed, patient’s weight, 2D or 3D PET acquisition mode, and other technical parameters and (sometimes) the patient’s compliance are mainly included. These data confirm that age and sex should be considered in assessing PET dose, as recommended by regulatory agencies such as the ICRP and Euratom [21, 22].

The mean risk reduction was 6.5% after applying ALGO in the study sample as a whole, 9.2% on average for female patients, and 20.3% for 18- to 29-year-olds. In this younger age bracket, it is crucial to reduce all potential exposure to radiation for medical purposes, particularly in females, as the cumulative effects of tests with ionizing radiation associated with a longer life expectancy multiply the already non-negligible initial risk. To give an example, an 18-year-old female with lymphoma who has undergone five FDG PET and diagnostic CT scans have been exposed to 10–15 mSv of ionizing radiation per scan, for a total of 50–75 mSv, with an ACR of up to 1.4 (1 in 71) [30]. In such a situation, even a fractional reduction in the PET dose may significantly reduce her considerable cancer risk. Direct evidence from human population studies shows that doses of 50–100 mSv (protracted exposure) or 10–50 mSv (acute exposure) raise the risk of developing secondary cancers later in life [16]. In the present study, consistently with the available literature, our ALGO reduced the ACR particularly for female patients [27]. The ALGO also lowered the ACR for patients aged 30–60 years.

Based on a time-dose equation, the ALGO inversely modified the FDG dose and PET scan time, making the former 20% lower and the latter 20% longer for younger patients, and vice versa for older patients. There were consequently no significant drawbacks in terms of session times or tracer doses. In the younger patients (who are generally more compliant), the 20% increase in PET scan time (corresponding to a few minutes longer) had negligible negative effects compared with the dosimetric advantages. In the older population (who tend to be less compliant and to experience comorbidities, pain or dyspnea), motion can be a significant cause of artefacts, particularly with longer scan times, so a 20% shorter scan time could have the advantage of reducing the motion artefacts that degrade PET images [5]. Given the known lengthy latency for the onset of new tumors, increasing older patients’ tracer dose has a negligible influence on their cancer risk because they are less liable to tumor induction and they have a shorter life expectancy. Conceptually, our data suggest an advantage of increasing the scan time to reduce the FDG dose in some younger patients (i.e., < 40 years), and females especially, and of doing the opposite in older patients (i.e., > 70 years), especially if male. This could be managed simply by knowing the sex and age of the patients scheduled for a given daily session, with no major effect on the time it takes and the amount of tracer needed.

The delicate issue of medical ionizing radiation must be addressed sensibly, given the broad spectrum of theories regarding the cancer risk from low-dose exposures [14–20]. Despite new PET and CT hardware and software being adopted in clinical practice [4–6, 12, 13], FDG PET/CT remains an imaging method that involves high doses of radiation, with PET accounting for about 50% of the total exposure [9, 11]. While many studies have focused on replacing CT with MRI for anatomical co-registration, fewer efforts have been made so far to reduce the injected radiotracer dose [11]. Major obstacles to reducing radiotracer doses include the increase in image noise, which affects diagnostic accuracy, or the need to extend the scan time, which adds to the workload. To overcome these issues, an artificial intelligence algorithm may be able to significantly reduce PET imaging time and radiation doses while maintaining image quality and SUV accuracy [30]. A different approach to reducing radiotracer doses while maintaining diagnostic accuracy was adopted in an Italian multicenter trial that proposed a segmental PET/CT approach in certain clinical subsets, to characterize solitary pulmonary nodules, for instance [7, 8]. The potential of the ALGO lies in the chance to obtain an immediate, customized reduction in the ACR, using the currently most popular hybrid scanners. As has been happening for cancer therapies, new hardware and software will converge towards an individual radiotracer dose that is as low as possible to extend the uses of PET.

The data reported here were retrospective estimates of the cancer risk, not records of real adverse events. That said, although the BEIR VII risk models used to estimate the excess cancer risk are imprecise and represent estimated risks extrapolated to the clinical setting from epidemiological data, this approach is widely used (because there are no alternative methods for assessing the potential risks of low-dose radiation) and provides the basis for regulatory systems [17, 31]. In our population, the positive effect of ALGO on overall cancer risk may seem negligible (given the high basic incidence of neoplasms), but it would seem logical to adopt it for such widely used diagnostic protocols as PET, in which case even a minimal risk reduction can have an impact. Reducing the risk for young people who will need repeated investigations with ionizing radiation is even more important (and demanded by national legislation), particularly if routinely used doses are higher than those described here.

Finally, it was mentioned earlier that using our ALGO did not carry any significant operative drawbacks, concerning the amount of the tracer needed, for instance. In fact, if patients whose dose is reduced by 20% weigh significantly less than those whose dose is increased, then the total amount of radiopharmaceutical needed may change. On the other hand, it is unlikely that all the younger people would be much lighter than all the older people needing more tracer.

## Conclusions

The present study found that age and sex are both decisive factors influencing the risk of radiation in patients undergoing PET imaging. Weight, age, and sex should be the “key points” for the identification of the appropriate dose for a given patient a priori; however, further prospective studies are mandatory. Our proposal may promote the use of a practical and easy strategy—like our ALGO protocol—that, by tailoring the dose of radiotracer to a patient’s age, was able to significantly reduce the overall biological risk associated with a session of PET. The benefit was particularly evident in young people and females, in line with the latest recommendations of the regulatory agencies and with national laws regarding the mandatory principle of optimization. This goal was achieved with no loss of the full benefits of PET/CT imaging, without affecting its diagnostic accuracy in individual patients, and without incurring any operative drawbacks in the daily workflow.

## Data Availability

Please contact the corresponding author for data requests.
